# Elevated fluoride concentration levels in rural villages of Siddipet, Telangana State, South India

**DOI:** 10.1016/j.dib.2017.11.088

**Published:** 2017-12-06

**Authors:** Adimalla Narsimha

**Affiliations:** Department of Applied Geochemistry, University College of Science, Osmania University, Hyderabad 500007, India

**Keywords:** Groundwater quality, Fluoride contamination, Siddipet region, Telangana State

## Abstract

Fluoride beyond desirable amounts(0.6–1.5 mg/L) in groundwater is a major problem and fluorosis is a very dangerous and deadly disease affecting millions of people across the World (Bell and Ludwig, 1970; Adimalla and Venkatayogi, 2017; Narsimha and Sudarshan, 2013, 2017a, 2017b) [1], [2], [3], [4], [5]. The investigated area is located in north-eastern part of Medak district, Telangana state and fluoride concentration in groundwater samples was measured by ion selective electrode method and its ranges from 0.4 to 2.2 mg/L with a mean value of 1.1 mg/L. Therefore, fluoride concentration data advised to the village people are consume drinking water which has less than 1.5 mg/L fluoride to avoid further fluorosis risks.

**Specifications Table**

**Table:** 

Subject area	*Earth Science*
More specific subject area	*Hydro-geochemistry*
Type of data	*Table and figure*
How data was acquired	*Thermo Scientific Orion Star A214 Benchtop pH/ISE meter*
Data format	*Analyzed*
Experimental factors	*Samples were collected in 1.0 l polyethylene bottles previously thoroughly cleansed with deionized water and subsequently with sampled groundwater before filling.*
Experimental features	*Determine the content levels of fluoride*
*Data source location*	*Location: Siddipet, Region: Medak, State: Telangana, India GPS: E longitude 78.76942–78.90232 and N latitude 18.06768–18.24402*
Data accessibility	*Data is with this article*

**Value of the data**

• Elevated fluoride concentration (> 1.5 mg/L) groundwater does not suitable for drinking purposes and if continuous ingest this water for a long period of time will surly effects on health especially, in children's and pregnant women.
• In the rural village people depends only on groundwater for drinking and house hold applications. Hence, finding of this study suggests to the residents that to drink water below maximum permissible limit (< 1.5 mg/L), to avoid further fluorosis problem in the villages.
• This data will surly helpful to scientific community those who work on this field of water quality, water pollution and also it is very informative for local NGO's and health policy makers to educate the rural people and protect from this deadly disease of fluorosis.

## 1 Data

In the Siddipet area of Medak region the concentration of fluoride in the groundwater is ranging from 0.4 to 0.9 mg/L, 1 to 1.4 mg/L, 1.5 to 1.9 mg/L and 2 to 2.2 mg/L (Table 1). As seen in , it shows that the relationship between fluoride and other chemical elements (Fig. 2) and silicate weathering processes is depicted in Fig. 3 [1], [2], [3], [4], [5].

**Fig. 1:**
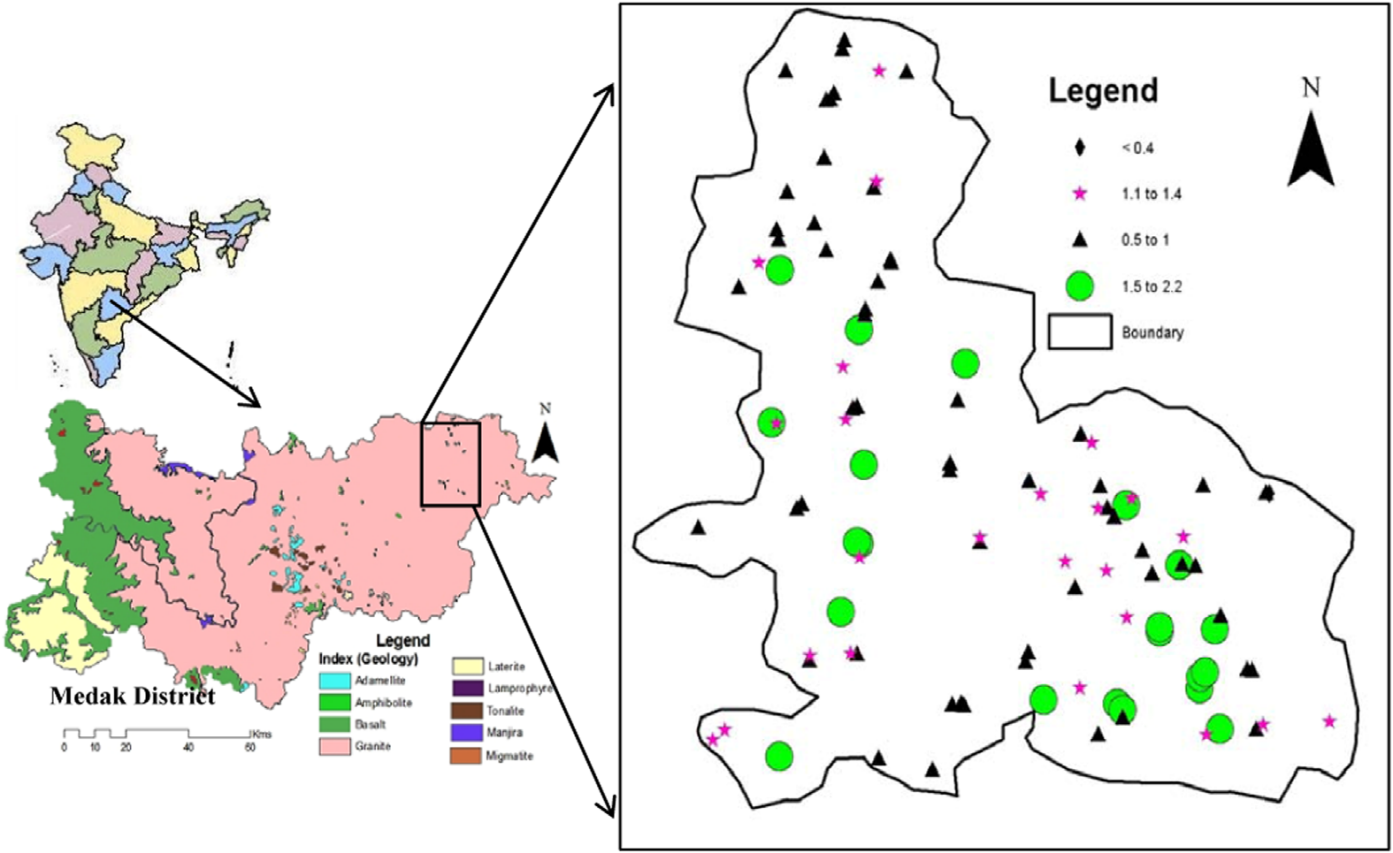
Study area showing location of wells sampled for groundwater analysis fluoride distribution in the Siddipet area.

**Fig. 2:**
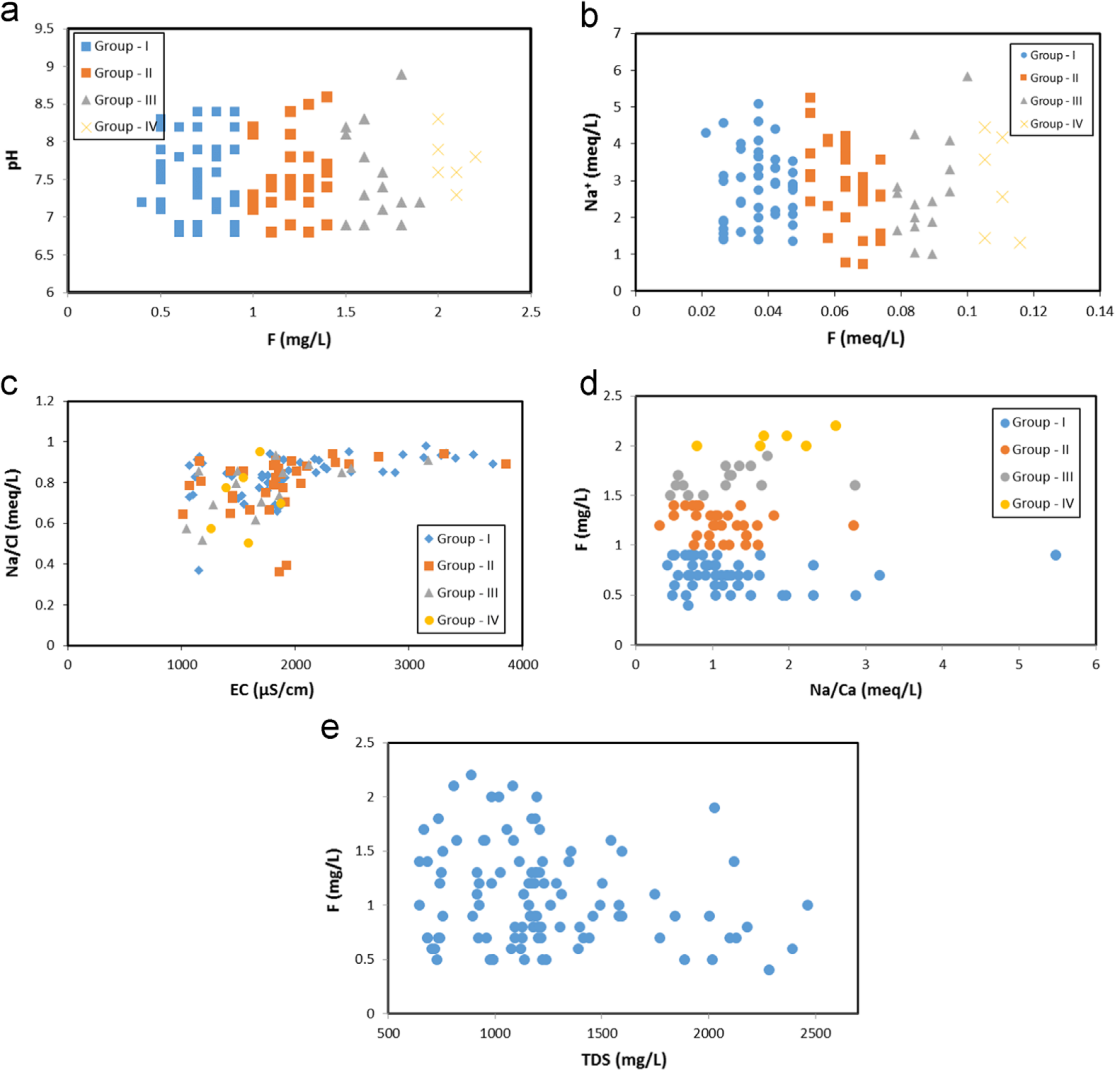
Relationship among fluoride and other elements in the groundwater of hard rock aquifers of Siddipet, Telangana State, India.

**Fig. 3:**
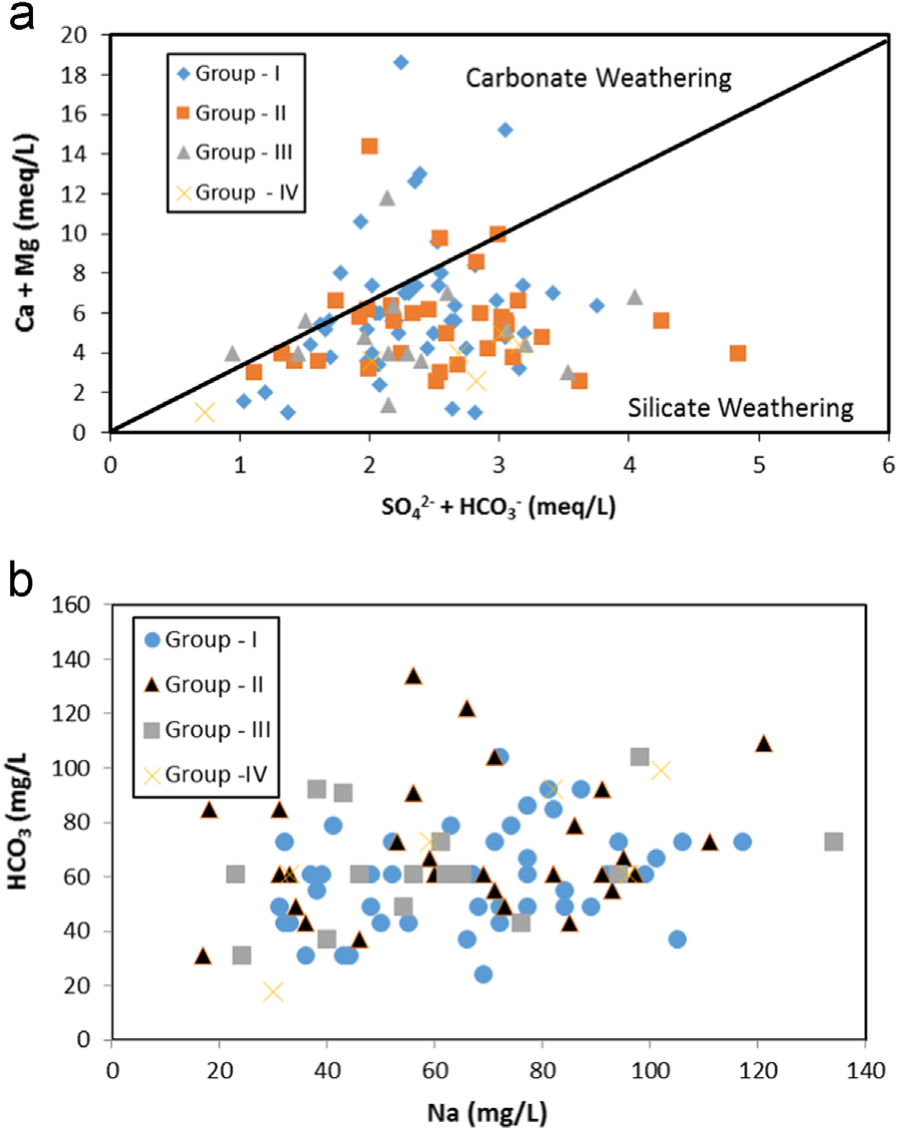
**a**, **b** Scatter diagram for carbonate weathering vs silicate weathering processes dissolution of rock salts and weathering of sodium bearing minerals.

**Table 1:** Descriptive statistics for F^-^ and other physicochemical parameters in the Siddipet area.

Parameters	pH	EC	TDS	TH	$HC{O_{3}}^{–}$	Cl^-^	$S{O_{4}}^{2-}$	$N{O_{3}}^{-}$	F^-^	Ca^2+^	Mg^2+^	Na^+^	K^+^
Min	6.8^a^	1070	684.8	65	24	25	25	9	0.4	10.02	6.075	31	1
	6.8^b^	1010	646.4	75	31	28	25	20	1	26.052	15.795	17	1
	6.9^c^	1040	665.6	60	31	57	21	22	1.5	14.028	8.505	23	1
	7.3^d^	1260	806.4	50	18	36	21	9	2	10.02	6.175	30	2
													
Max	8.4^a^	3740	2393.6	565	104	973	108	361	0.9	186.372	112.995	117	61
	8.6^b^	3850	2464	415	134	746	156	321	1.4	144.288	87.48	121	85
	8.3^c^	3170	2028.8	330	104	511	137	194	1.9	118.236	71.685	134	10
	8.9^d^	1870	1196.8	225	99	675	97	198	2.2	50.1	30.375	102	4
													
Mean	7.5^a^	2020.4	1293.06	218.08	59.62	254.64	63.84	123.6	0.706	61.48	37.28	65.96	7.96
	7.5^b^	1866.06	1194.28	213.76	68.94	220.21	69.94	104.35	1.20	54.78	33.21	65.45	6.85
	7.6^c^	1782.67	1140.91	179.67	63.93	188.20	63.67	88	1.66	50.77	30.78	60.93	4.20
	7.5^d^	1556.67	996.27	159.17	67.33	199.67	62.50	71.13	2.07	34.40	20.86	67.00	2.83
													
Median	7.4^a^	1860	1190.4	200	61	207.5	56	110	0.7	56.112	34.02	67	4
	7.4^b^	1830	1171.2	210	61	170	65	79.2	1.2	54.108	32.805	65	4
	7.4^c^	1700	1088	175	61	128	57	66	1.6	44.088	26.73	56	4
	7.6^d^	1565	1001.6	182.5	67	90.5	64.5	41.8	2.05	38.076	23.085	70.5	3
													
Std Dev	0.51^a^	711.94	455.64	101.29	17.32	186.75	24.43	85.20	0.14	34.14	20.70	22.01	12.46
	0.62^b^	580.73	371.67	69.96	20.38	147.35	33.29	55.48	0.12	23.82	14.44	29.80	2.57
	0.62^c^	580.73	371.67	69.96	20.38	147.35	33.29	55.48	0.12	23.82	14.44	29.80	2.57
	0.47^d^	215.93	138.20	64.92	28.84	249.22	27.35	75.57	0.08	14.42	8.74	31.24	0.75

EC is expressed as µS/cm, and all other parameters are expressed as mg/L.

a Group – I,

b Group – II,

c Group – III,

d Group – IV

## 2 Experimental design, materials, and methods

### 2.1 The study area description

Siddipet is situated about 105 km north of Hyderabad on Hyderabad–Karimnagar State highway, and is bounded by E longitude 78.76942–78.90232 and N latitude 18.06768–18.24402. The area under investigation falls under semi-arid zone, with a hot, humid climate, and predominantly occupied by granite/gneiss of Archean age (Fig. 1).

### 2.2 Sample collection and analytical procedures

104 groundwater samples were collected from 39 villages of Siddipet region in the month of July 2014. The fluoride concentration in groundwater was determined electrochemically, using Thermo Scientific Orion Star A214 Benchtop pH/ISE meter, using the USEP ion selective electrode method. As per experimental requirement, 2 ml of total ionic strength adjusting buffer grade III (TISAB III) was added in 20 ml of groundwater sample and determined the fluoride concentration. Calcium, magnesium, chloride, carbonate and bicarbonate were analyzed by a titration method. Sodium and potassium concentrations were determined using a flame photometer (Systronics, 130). Sulphate and nitrate were determined using a UV–visible spectrophotometer (Spectronic, 21, BAUSCH and LOMB). Using pH/EC/TDS meter (Hanna HI 9811-5), the EC, pH and TDS of water samples were measured.
